# A pilot study of helicopter emergency medical services in the Zhoushan Archipelago: clinical experience and operational insights from 18 cases

**DOI:** 10.3389/fpubh.2026.1834164

**Published:** 2026-08-06

**Authors:** Yongjun Zheng, Ziyun Lin, Junyan Wang, Liyang Chen, Zhoubo Wang, Yuechuan Shen

**Affiliations:** Emergency Department, Zhoushan Hospital of Zhejiang Province, Zhoushan, Zhejiang, China

**Keywords:** acute coronary syndrome, helicopter emergency medical services (HEMS), maritime rescue, remote islands, stroke

## Abstract

**Background:**

The Zhoushan Archipelago faces challenges in delivering timely emergency care due to its dispersed geography and centralized medical resources. To address delays associated with ferry transport for critically ill patients on remote islands, a maritime helicopter emergency medical service (HEMS) was established.

**Methods:**

We conducted a pilot, retrospective study of all patients transported by this novel HEMS between March 2024 and June 2025. Data from electronic medical records were analyzed to assess initial feasibility, describe patient demographics, in-flight interventions, and clinical outcomes.

**Results:**

In this initial case series of 18 patients, the majority were male (*n* = 17) with conditions split evenly between traumatic injuries (*n* = 9) and acute medical events (*n* = 9). HEMS demonstrated the capability for rapid transfer and advanced in-flight care. Preliminary outcomes indicated that 17 of 18 patients survived to discharge, with several receiving critical surgery within 1 h of arrival. One death occurred in a patient with a severe stroke.

**Conclusion:**

This pilot study suggests that maritime HEMS is a feasible and promising intervention for improving emergency response in remote island settings, with the potential to significantly reduce transfer times. Our initial experience supports the need for larger-scale studies to conclusively determine its impact on patient outcomes. Further improvements in operational efficiency are warranted.

## Introduction

The Zhoushan Archipelago, the largest archipelago in China, consisting of 1,390 islands, exhibits a signxificant spatial imbalance in the distribution of medical resources, with resources primarily concentrated on the main island, while the remote islands suffer from a severe lack of medical infrastructure. Residents of the remote islands rely heavily on ferries for transportation, and the lack of convenient transportation significantly limits the timely treatment of critically ill patients in these areas.

As an international tourist destination, Zhoushan receives approximately 100 million visitors annually, with the majority concentrated on the remote islands, such as Putuo Mountain, Shengsi Islands, and Dongji Island. The health centers in these areas are limited by equipment and technical capabilities, often unable to provide comprehensive treatment. Patients must be transported by ferry to tertiary hospitals on the main island, which frequently causes delays in the “golden hour” for trauma care (ACS-COT, 2024), leading to poor patient outcomes.

Additionally, as one of the four major fishing grounds in the world, Zhoushan has a large-scale marine fishing industry, accompanied by a high incidence of occupational injuries at sea, such as trauma and drowning. Historically, due to the inefficiency of ferry transport, the mortality and disability rates among injured individuals in remote maritime areas have been high. Therefore, there is an urgent need for a new transportation method to reduce the treatment time for patients with cardiovascular accidents or trauma occurring on remote islands.

Against this background, in 2024, the Zhejiang Provincial Emergency Management Bureau, in collaboration with the Zhejiang Provincial Emergency Management Aviation Rescue Center, Zhoushan Municipal Emergency Management Bureau, Zhoushan Health and Family Planning Commission, and Zhoushan Hospital, formed a maritime air rescue team to provide rapid transportation and treatment for patients in offshore and remote areas. This study presents a statistical analysis of patients transported by helicopter to our hospital between March 2024 and June 2025, aiming to evaluate the impact of the maritime air rescue team on the treatment outcomes of patients from remote areas.

## Patients and methods

Ethical approval for this retrospective study was granted by the Institutional Review Board of Zhoushan Hospital Ethics Committee (Approval No: 2025-016-001) on (Date of 2025.1). The approval included a waiver of informed consent and granted retrospective permission for the analysis of all data from the study period [(2024.3)–(2025.6)], as the research involved no more than minimal risk and all patient data were anonymized.

Helicopter configuration: H225 helicopter load and performance data: empty weight: 5543 kg. Maximum payload: 5457 kg. Maximum takeoff weight: 11 tons. External sling load: 4750 kg. Standard fuel tank: 2588 liters. Maximum fuel capacity: 2908 liters. Maximum airspeed (VNE): 324 km/h. Cruise speed (6000 feet): 262 km/h. Maximum range: 1197 km. Maximum endurance: 6 h and 16 min. Personnel Configuration: divided into two parts: Zhejiang Provincial Emergency Management Aviation Rescue Team: 2 helicopter pilots, 1 navigator, and 2 hoist rescue operators. Zhoushan Hospital's Medical Department is responsible for coordination, with the Emergency Department as the lead department, assigning 1 physician and 1 nurse to participate in the rescue treatment.

Rescue operations take place from 6:00 a.m.−5:00 p.m. Due to the helicopter's configuration, night flights are not feasible.

This was a single-center, retrospective, pilot study designed to evaluate the initial implementation and feasibility of a newly established maritime HEMS. Given the novelty of this service in our region and the anticipated low initial case volume, a formal sample size calculation was not performed *a priori*. The study aimed to capture all consecutive cases during the initial operational period to generate hypotheses and inform future research.

## Results

**Figure 1:**
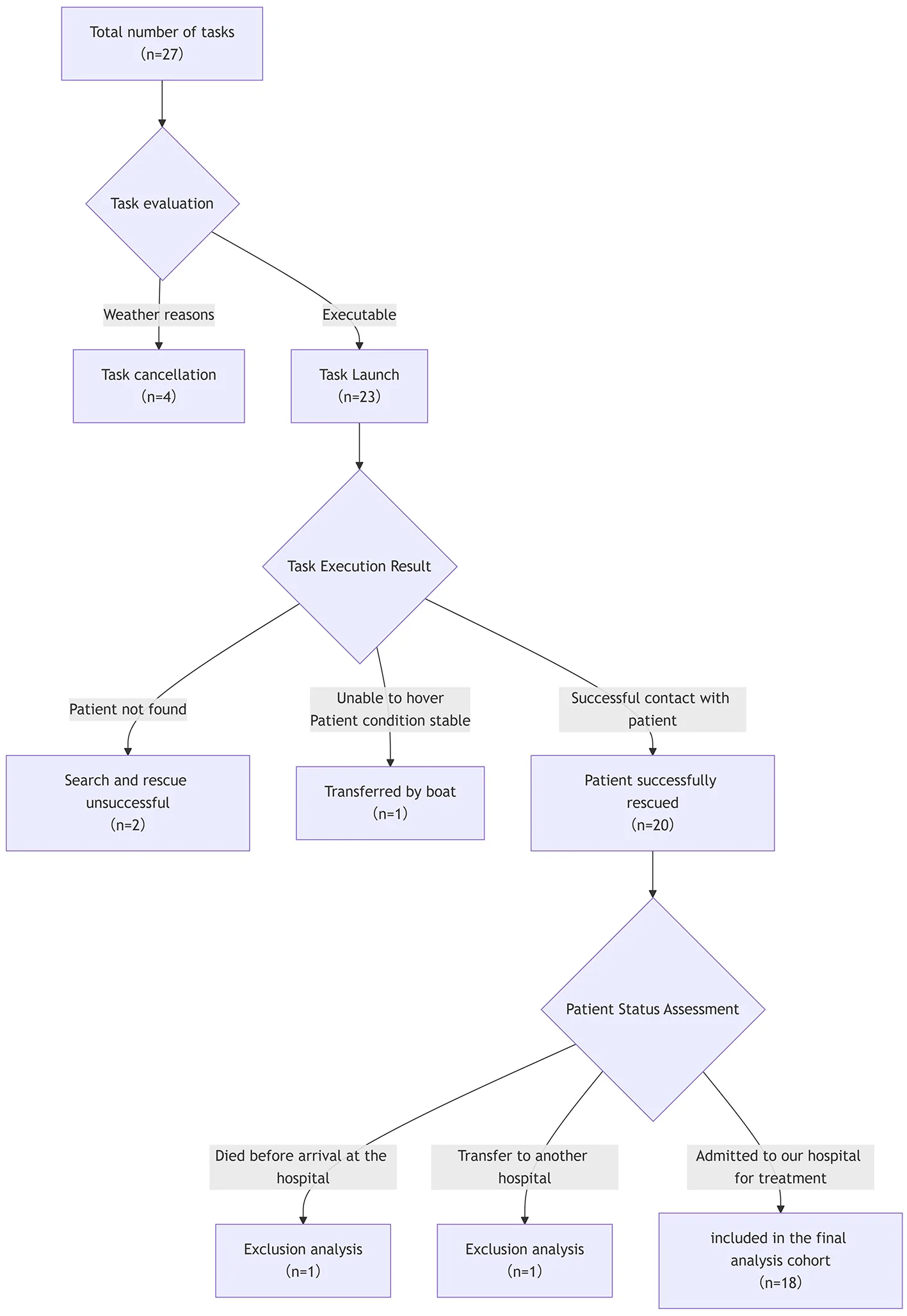
Consort patient flowchart.

The study cohort comprised 18 patients successfully transported by maritime HEMS (Figure 1 and Supplementary Table 1), with baseline characteristics and overall outcomes summarized in Table 1. The median age was 51 years (IQR 42.5–55.8) and the population was predominantly male (94.4%). Cases were equally divided between trauma (50%) and spontaneous medical emergencies (50%); the nine trauma patients had a median Injury Severity Score of 5 (IQR 3–14, moderate severity), and the overall median admission Glasgow Coma Scale was 15 (IQR 8.3–15). Mission timelines showed a median onset-to-dispatch interval of 360 min (IQR 97.5–660), a total prehospital time of 240 min (IQR 180–480) from onset to hospital arrival, and a maritime transfer phase lasting 120 min (IQR 60–180). In-hospital, 55.6% required surgery, median ICU stay was 0 days (IQR 0–4.3), and median total hospital stay was 15.5 days (IQR 9.5–23.5). At discharge, 94.5% survived −66.7% achieved full recovery, 27.8% survived with impairment, and 5.6% died during hospitalization.

**Table 1:** Baseline characteristics, mission timelines, and outcomes of the 18-patient cohort.

Characteristic	Value (*N* = 18)
Demographics	
Age (years), median (IQR)	51 (42.5, 55.8)
Male sex, n (%)	17 (94.4)
Clinical presentation	
Etiology, n (%)	
Trauma	9 (50.0)
Spontaneous (Stroke, ACS, etc.)	9 (50.0)
Final Diagnosis, *n* (%)	
Traumatic brain injury	3 (16.7)
Spontaneous intracranial hemorrhage	4 (22.2)
Acute coronary syndrome	3 (16.7)
Extremity/soft tissue trauma	5 (27.8)
Other (cardiac arrest, multisystem trauma)	3 (16.7)
ISS, median (IQR), n=9	5 (3, 14)
GCS on admission, median (IQR)	15 (8.3, 15)
Mission times (minutes), median (IQR)	
Onset to helicopter dispatch	360 (97.5, 660)
Total prehospital time	240 (180, 480)
Maritime transfer time	120 (60, 180)
Treatment & outcomes	
Underwent surgery, n (%)	10 (55.6)
ICU length of stay (days), median (IQR)	0 (0, 4.3)
Total hospital stay (days), median (IQR)	15.5 (9.5, 23.5)
Discharge condition, n (%)	
Survived with full recovery	12 (66.7)
Survived with impairment	5 (27.8)
Deceased	1 (5.6)

IQR, interquartile range; ISS, Injury Severity Score; GCS, Glasgow Coma Scale; ACS, Acute Coronary Syndrome; ICU, Intensive Care Unit.

Case 1: a 45-year-old male was transported via helicopter to our hospital due to “head injury caused by a cable strike with impaired consciousness for 6 h.” The patient was struck in the head and face by a fractured cable while working on a deep-sea fishing vessel, resulting in immediate confusion, active bleeding from the head and facial regions, and oozing of blood from the mouth and nose. Upon discovery by colleagues, emergency assistance was promptly requested. The specialized maritime helicopter rescue team immediately applied for urgent airspace authorization. The total duration from the initial distress call to hospital admission via helicopter was 6 h.

In-flight medical management: the patient underwent primary trauma assessment according to the ABCDE protocol. He presented with impaired consciousness (GCS score: E2V1M2, total 5), anisocoria (right pupil 5 mm, left pupil 4 mm, both with sluggish light reflex), and an 8 cm laceration on the head with active bleeding, accompanied by bloody secretions from the mouth and nose. No deformities or open injuries were observed in the chest, abdomen, or extremities. Findings were suggestive of moderate to severe traumatic brain injury.

Vital signs monitoring: temperature 36.3 °C, BP 187/102 mmHg, HR 113 bpm, RR 23 bpm, SpO_2_ 92%.

Emergency interventions: spinal immobilization with a cervical collar, hemostatic compression dressing of the head wound, placement of a nasopharyngeal airway, establishment of two intravenous lines, administration of 250 mL mannitol infusion for intracranial pressure reduction, oxygen therapy, pre-collection of blood samples, and continuous electrocardiographic monitoring. Vital signs and clinical changes were transmitted in real time via a 5G-based telemedicine system. Given the high suspicion of severe traumatic brain injury with signs of cerebral herniation, the neurosurgery team was alerted for potential emergency surgery.

**Figure 2:**
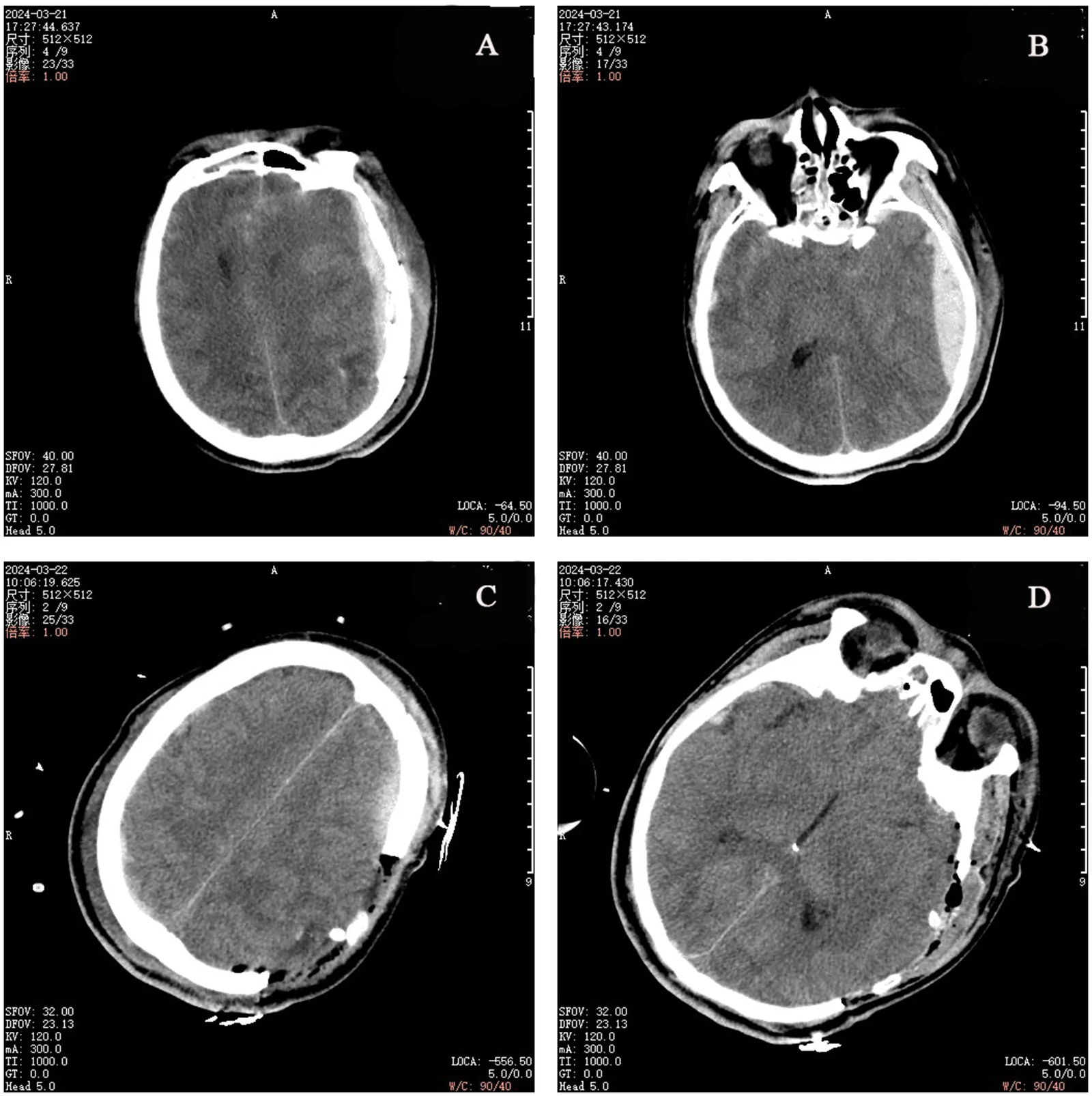
Preoperative and postoperative cranial CT images of Patient 1 with traumatic brain injury. **(A, B)** Preoperative CT scans showing midline shift, cerebral swelling, herniation, left temporal bone fracture, and cerebral contusion. Traumatic subdural and epidural hemorrhages are also evident. **(C, D)** Postoperative CT scans following decompressive craniectomy, demonstrating resolution of herniation and alleviated cerebral edema compared to preoperative findings.

Post-admission course: upon arrival, vital signs were: T 36.5 °C, HR 119 bpm, RR 20 bpm, BP 198/110 mmHg, SpO_2_ 97% (pre-intubation). Physical examination revealed coma (GCS E1V1M1, total 3), bilateral dilated pupils, and absent light reflexes. Immediate endotracheal intubation with mechanical ventilation was performed. Therapy was intensified with continued dehydration, intravenous infusion of urapidil 150 mg via syringe pump for blood pressure control, and tranexamic acid for hemostasis. Cranial CT confirmed isolated severe intracranial injury without extracranial hemorrhage (Figure 2). Decompressive craniectomy was performed within 1 h of admission. The patient was successfully weaned from mechanical ventilation on postoperative day 7 and transferred to a general ward.

Despite aggressive resuscitation, the total prehospital time exceeded 6 h, surpassing the golden hour for trauma care. This delay resulted in irreversible brainstem injury. At discharge, the patient remained comatose (GCS 5) and was transferred to a rehabilitation facility. In comparable cases of severe traumatic brain injury, prolonged transport by ship often leads to mortality during transit. In this case, however, helicopter evacuation significantly shortened transfer time, enabling timely surgical intervention and ultimately saving the patient's life.

Case 2: a 54-year-old male was transferred to our hospital via helicopter due to “left flank and abdominal pain for 4 h after being struck by a cable.” The injury occurred when the patient was hit by a snapped cable during fishing operations, resulting in severe left flank and abdominal pain. There was no reported loss of consciousness, headache, chest pain, dyspnea, or limb injury. After being found by fellow crew members, emergency assistance was requested. The total time from the distress call to hospital admission via helicopter was 4 h.

In-flight medical management: primary trauma assessment (ABCDE protocol) revealed clear consciousness, pallor, and coherent speech. No injuries were observed in the head or neck. Left-sided chest and abdominal swelling with bruising were noted, without significant external bleeding. Abdominal examination showed generalized muscle guarding, with tenderness and rebound tenderness over the left upper quadrant, and dullness on percussion. Pelvic compression test was negative, and limb movement was normal. Suspected diagnosis included solid organ injury (spleen/kidney) with retroperitoneal hematoma.

Vital signs were as follows: temperature 36.2 °C, heart rate 119 bpm, respiratory rate 28 bpm, blood pressure 77/45 mmHg, and SpO_2_ 93%. Interventions included chest and abdominal binder application, establishment of two large-bore intravenous lines, infusion of 1000 mL compound sodium chloride solution to maintain perfusion (target SBP >80 mmHg), pre-collection of blood samples, continuous ECG monitoring, and intramuscular administration of 100 mg tramadol for analgesia.

**Figure 3:**
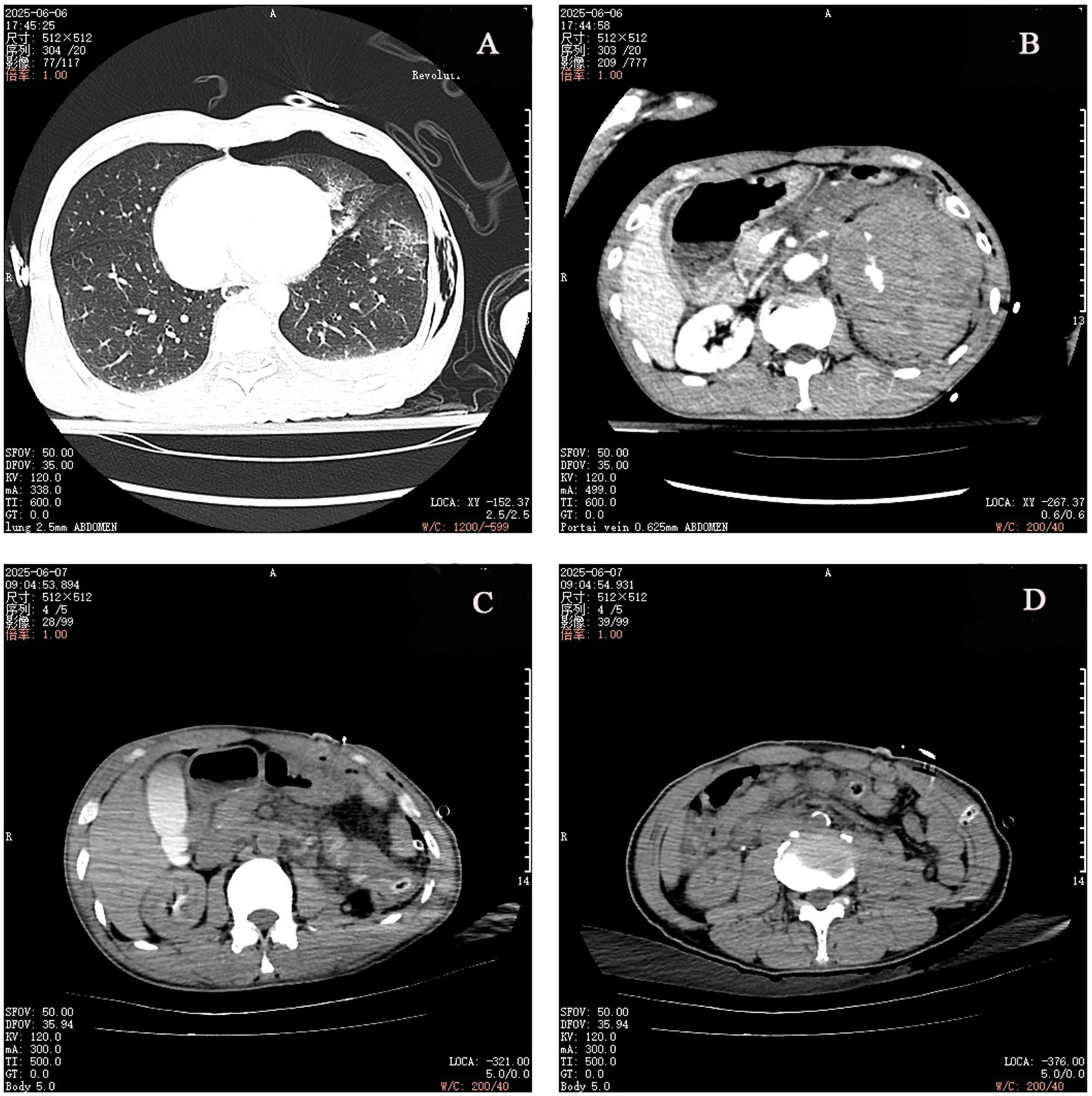
Preoperative and postoperative CT images of the chest and abdomen in Patient 2. **(A)** Chest CT scan reveals traumatic wet lung, hemopneumothorax, and multiple rib fractures. **(B)** Abdominal CT scan shows a left renal contusion with a large associated hematoma. **(C, D)** Follow-up abdominal CT scans after left nephrectomy demonstrate significant resolution of the hematoma compared to preoperative findings.

Post-admission course: upon admission, vital signs were: T 36.1 °C, HR 107 bpm, RR 22 bpm, BP 109/65 mmHg, SpO_2_ 95% (pre-intubation). Physical examination indicated clear consciousness, pallor, coherent speech, and no head or neck injury. Left chest and abdominal swelling with bruising and significant tenderness were observed, with normal limb mobility. Contrast-enhanced chest CT (Figure 3A) and abdominal CT (Figures 3B–D) were performed. Laboratory results indicated renal impairment: creatinine 139.9 μmol/L, and estimated glomerular filtration rate (eGFR) 49.09 mL/min/1.73m^2^ (CKD stage 3a). Following discussion by the multidisciplinary trauma team, the patient was diagnosed with shattered left kidney, rib fractures, and hemopneumothorax. Immediate surgical intervention by the urology department was recommended, and blood products were prepared urgently. The patient underwent emergency exploratory laparotomy within 1 h of admission.

Surgical and postoperative outcomes: intraoperative findings confirmed shattered left kidney, splenic rupture, and diaphragmatic rupture. The procedures performed included left nephrectomy, splenorrhaphy, diaphragmatic repair, and left closed thoracic drainage. Intraoperative blood loss was approximately 2000 mL, requiring transfusion of 2 units of suspended red blood cells, 1000 mL of autologous blood salvage, and 350 mL of fresh frozen plasma.

The patient was transferred to the ICU postoperatively. Follow-up chest and abdominal CT on postoperative day (POD) 1 evaluated hematoma/effusion and thoracic drainage. The patient was successfully extubated on POD 2, transferred to a general ward on POD 4 upon stabilization, and discharged on POD 21. At discharge, the patient had chronic kidney disease (CKD stage 2) with creatinine 114 μmol/L and eGFR 63 mL/min/1.73m^2^ (improved from admission but not returned to baseline).

Case 3: a 54-year-old male was admitted via helicopter transfer due to “sudden loss of consciousness for 150 min.” The patient collapsed while walking, became unresponsive, and fell to the ground without limb convulsions or incontinence. Bystanders immediately called emergency services. Paramedics arrived within 5 min and confirmed cardiac arrest, initiating cardiopulmonary resuscitation and assisted ventilation. The patient was transported to a local hospital within 10 min of onset but remained without spontaneous circulation. Ventricular fibrillation occurred at 20 min post-onset, and one AED shock was delivered. Return of spontaneous circulation was achieved at 40 min post-onset, after which the local hospital requested helicopter transfer to our institution for further care. The total time from the request to hospital admission was 2 h.

In-flight medical management: assessment revealed coma (GCS score 3), pupils equal and round (3 mm diameter) with sluggish light reflexes. Vital signs included temperature 36.0 °C, heart rate 70 bpm, respiratory rate 18 bpm (bag-valve-mask ventilated), blood pressure 211/120 mmHg, and SpO_2_ 99%. Interventions included endotracheal intubation with bag ventilation, establishment of intravenous access, pre-collection of blood samples, and continuous ECG monitoring. During transport, the patient experienced vomiting with incontinence, requiring repeated airway suctioning to maintain patency. Suspecting acute coronary syndrome, the cardiology team was alerted via 5G telemedicine to await the patient's arrival.

Post-admission course: upon admission, vital signs were: T 36.2 °C, HR 67 bpm, RR 20 bpm, BP 132/86 mmHg, SpO_2_ 95%. Physical examination showed coma (GCS 3), pupils equal and round (3 mm) with sluggish light reflexes. Immediate bedside ECG revealed sinus tachycardia, complete right bundle branch block, and abnormal Q waves in inferior and anterolateral leads. Bedside echocardiography demonstrated left ventricular enlargement, wall motion abnormalities, and reduced systolic function. Laboratory results included troponin 0.393 ng/mL, myoglobin 535 ng/mL, CK-MB 46.44 ng/mL, and lactate 7.36 mmol/L. After evaluation by the cardiology team, a diagnosis of acute coronary syndrome was confirmed, and emergency percutaneous coronary intervention (PCI) was recommended. The patient was taken to the catheterization laboratory within 1 h of admission.

Intervention and postoperative outcomes: emergency PCI revealed proximal occlusion of the intermediate branch. Despite multiple attempts, the lesion could not be crossed with a balloon, and the procedure was concluded. The patient was transferred to the ICU for postoperative care. Successful extubation occurred on postoperative day (POD) 1. The patient was transferred to a general ward on POD 4 following stabilization and was discharged on POD 12 in improved condition.

Case 4: a 61-year-old male was admitted via helicopter transfer due to “sudden loss of consciousness for 4 h.” The patient experienced an abrupt, unexplained loss of consciousness 4 h prior to admission. 2 h after onset, family members brought him to a local hospital. At the local hospital, vital signs were: temperature 36.3 °C, heart rate 77 bpm, respiratory rate 19 bpm, blood pressure 213/121 mmHg, SpO_2_ 95%. Neurological assessment revealed coma with a Glasgow Coma Scale (GCS) score of 8 (E2V2M4). A preliminary diagnosis of cerebrovascular accident was made. Given the limited treatment capabilities at the local facility, symptomatic management was initiated, including intravenous omeprazole 40 mg for gastric protection and urapidil 150 mg via syringe pump for blood pressure control. Helicopter evacuation to our hospital was immediately requested. The total time from the rescue request to admission was 4 h.

In-flight medical management: during transport, the patient remained comatose (GCS 8). Vital signs were temperature 36.5 °C, heart rate 89 bpm, respiratory rate 19 bpm, blood pressure 207/116 mmHg, and SpO_2_ 94%. An oropharyngeal airway was placed to maintain patency, intravenous access established, blood samples pre-collected, and continuous ECG monitoring performed. En route, the patient experienced vomiting and incontinence, requiring oronasal suctioning. Using 5G technology, vital signs and case information were transmitted in real time to the receiving hospital, prompting neurology and neurosurgery teams to standby in the emergency room. Total time from symptom onset to arrival was approximately 4 h.

**Figure 4:**
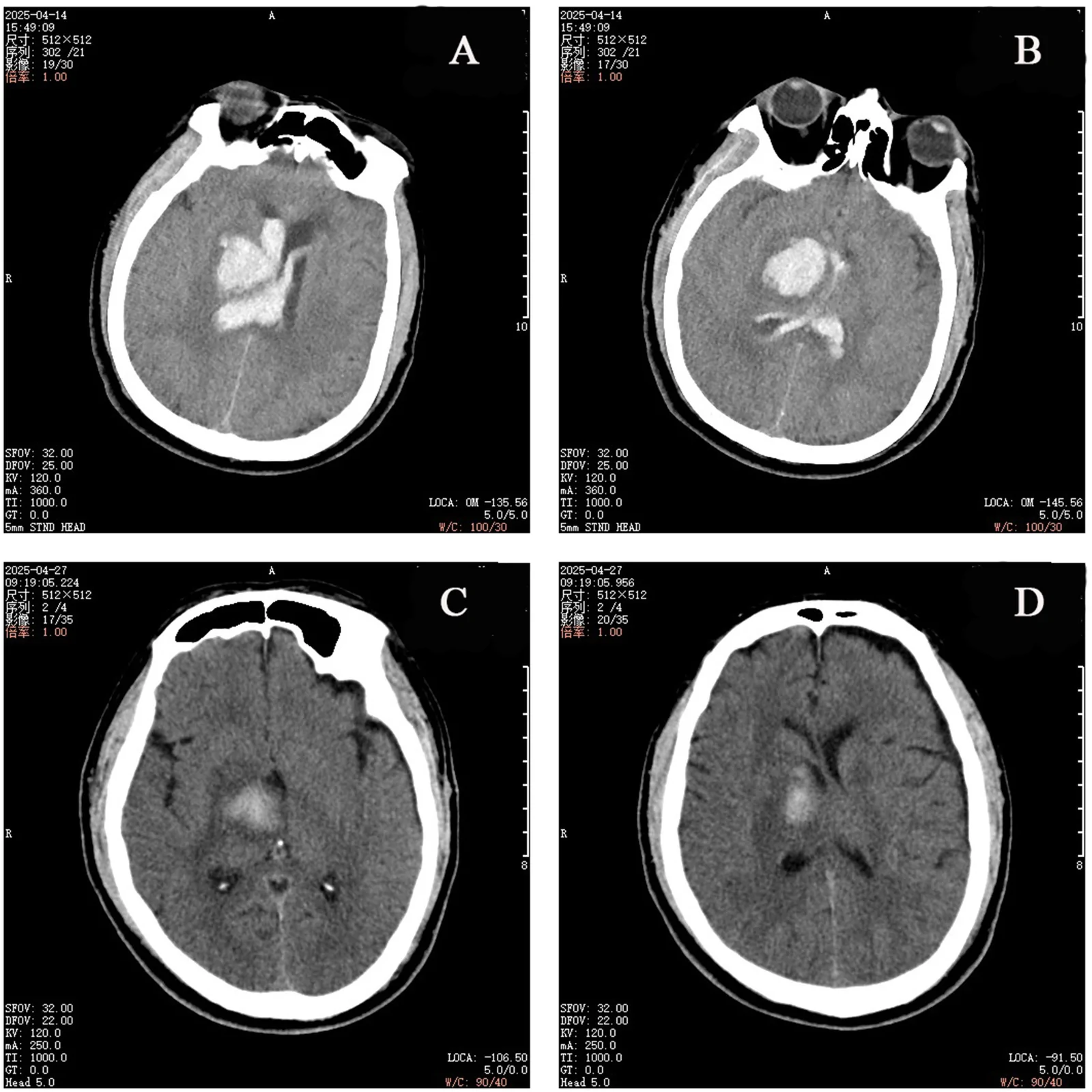
Preoperative and postoperative cranial CT images of Patient 4 with massive intraventricular hemorrhage. **(A, B)** Preoperative CT scans demonstrate massive intracerebral hemorrhage with ventricular extension and significant cerebral swelling. **(C, D)** Postoperative CT scans following hematoma evacuation and external ventricular drainage show successful removal of the hematoma and marked improvement of cerebral edema.

Post-admission course: upon admission, vital signs were temperature 36.2 °C, heart rate 71 bpm, respiratory rate 18 bpm, blood pressure 219/128 mmHg, and SpO_2_ 98%. Physical examination showed coma (GCS 4), anisocoria (left pupil 2 mm, right pupil 3 mm), and absent light reflexes. Protective endotracheal intubation with mechanical ventilation was performed immediately. Head CT imaging was obtained (see Figures 4A–D).

Intervention and outcomes: after neurosurgical consultation, emergency surgery was recommended. The patient was taken to the operating room within 1 h of admission, where intracranial hematoma evacuation was performed. The patient was transferred to a rehabilitation hospital on postoperative day 11 for further management. At discharge, the patient was in a state of drowsiness with a GCS score of 11.

Case 5: a 51-year-old male was admitted via helicopter transfer due to “sudden head discomfort for 3 h, and impaired consciousness for over 1 h.” The patient experienced sudden onset of head discomfort 3 h prior without an obvious cause and went to his room to rest. Approximately 30 min later, a coworker found him diaphoretic, able to protrude his tongue, with left-sided limb weakness and normal right-sided limb function. Helicopter rescue was immediately requested. While awaiting rescue, the patient vomited once, experienced urinary incontinence, and had one episode of convulsions lasting about 3 min. Around 2 h after symptom onset, the patient lost consciousness and became unresponsive. The total time from the rescue request to hospital admission was 3 h.

In-flight medical management: assessment during transport revealed coma (Glasgow Coma Scale score 5, E1V1M3). Vital signs included temperature 35.6 °C, heart rate 59 bpm, respiratory rate 13 bpm, blood pressure 197/103 mmHg, and SpO_2_ 89%. Management included placement of an oropharyngeal airway, supplemental oxygen, establishment of intravenous access, pre-collection of blood samples, and continuous ECG monitoring. Utilizing 5G technology, vital signs were transmitted, and a presumptive diagnosis of cerebrovascular accident was made. Urapidil (100 mg via syringe pump) was administered for hypertension. The neurosurgery and neurology departments were alerted to standby in the emergency room. 10 min prior to hospital arrival, the patient experienced sudden cardiac and respiratory arrest. Immediate cardiopulmonary resuscitation (CPR) was initiated onboard, along with administration of epinephrine (1 mg IV q4min, total 2 doses), resulting in brief return of spontaneous circulation (ROSC).

Post-admission course: upon admission, vital signs were: temperature 35 °C, pulse 0 bpm, respiratory rate 0 bpm, blood pressure unmeasurable, SpO_2_ unmeasurable. Physical examination revealed coma (GCS 3), bilaterally fixed and dilated pupils (6 mm diameter), cloudy corneas, non-palpable carotid pulses, apnea, and cyanotic lips. Arterial blood gas analysis showed pH 6.99, PO_2_ 12 mmHg, PCO_2_ 97 mmHg, lactate 10.60 mmol/L. Laboratory tests revealed WBC 16.0 × 10^9^/L and CRP 1.60 mg/L. Protective endotracheal intubation with mechanical ventilation was performed immediately.

Resuscitation and outcome: continuous chest compressions and administration of epinephrine (1 mg IV q4min, total 8 doses) were performed. After 30 min of sustained advanced cardiac life support, the patient was declared deceased.

## Discussion

This pilot study describes the initial experience and operational feasibility of implementing a maritime HEMS in the challenging geography of the Zhoushan Archipelago. While limited by a small sample size, our findings provide preliminary evidence that HEMS can overcome geographical barriers and facilitate the transfer of critically ill patients from remote islands. The observed flight times suggest a potential time saving compared to traditional ground or maritime transport, although direct comparisons were not feasible without baseline control data. However, a critical finding was the highly variable and often prolonged interval from symptom onset to helicopter dispatch (median 360 min, IQR 97.5–660), which highlights the most significant and adjustable bottleneck in the care pathway. This heterogeneity likely stems from multiple factors, including delayed patient recognition on remote islands, logistical challenges in patient extraction from vessels, and, as noted in our case series, delays in coordination and ATC clearance. Therefore, while these early results generate the hypothesis that such a system may reduce in-flight transit times, the substantial pre-dispatch delays underscore that the overall benefit is contingent on optimizing the entire rescue chain. Prehospital teams proficiently delivered advanced life support—including airway management, dual intravenous access, and continuous monitoring—while utilizing 5G technology for remote consultation and pre-arrival hospital preparation (1, 3, 4). In this series, the majority of transported patients showed favorable recovery, with several undergoing surgery within 1 h of arrival. However, these outcomes are observational and cannot be causally attributed to HEMS alone, given the absence of a control group.

However, the rescue process also revealed persistent challenges, as evidenced by the single mortality case within our cohort (Patient 5). This case offers a critical lesson: the primary cause of death was an extremely severe and rapidly progressing condition, highly consistent with a massive stroke (5). It involved substantial delays—a roughly 30-min lag in the rescue call, 40 min for air traffic control (ATC) clearance and crew mobilization, and a total prehospital time of 3 h—well beyond the optimal intervention window (5). Although cardiopulmonary arrest occurred in-flight and brief return of spontaneous circulation (ROSC) was achieved, the confined cabin space and lack of portable imaging limited sustained advanced life support and early definitive diagnosis. Upon arrival, the patient presented with irreversible brain injury, severe acidosis, and multi-organ failure, leading to unsuccessful resuscitation despite maximal effort (6). This outcome highlights that while HEMS offers rapid transport, reversing outcomes in time-critical pathologies requires both early effective medical intervention and robust in-flight advanced life support capabilities.

The analysis of timelines and the variability therein directly informs the necessary operational improvements. HEMS currently faces multiple operational constraints, with prolonged ATC clearance being a major bottleneck (1). Future research should evaluate the feasibility and cost-effectiveness of an Emergency Green Channel with ATC to potentially shorten clearance time, as well as the establishment of satellite forward operating bases (FOBs) in outer islands to accelerate response. Furthermore, public education campaigns on remote islands to improve early recognition of medical emergencies are crucial to address the patient-dependent component of the delay. To enhance treatment efficacy and safety, the concept of upgrading HEMS to a dedicated “Airborne Resuscitation Unit”—equipped with focused assessment with sonography for trauma (FAST) ultrasound, blood products, advanced respiratory support, and reinforced equipment restraints—warrants further investigation. Similarly, the development of night-flight capability represents an important future direction for achieving 24/7 operational readiness and expanded coverage (7).

Technologically, integrating 5G with satellite communications could enable real-time vital sign transmission and remote guidance in maritime settings (8); future studies should assess the impact of such integration on rescue efficiency and operational outcomes. High operational costs remain a barrier to accessibility, and health-economic evaluations are needed to determine the viability of incorporating HEMS into public medical insurance or aid systems.

Finally, future research should examine the impact of high-frequency simulation training for medical crews under realistic conditions (e.g., noise and vibration) on operational performance (2, 9), as well as the efficacy of robust psychological support systems in safeguarding rescue personnel's mental wellbeing in high-stress environments (10).

The most significant limitation of this study is its small sample size (*n* = 18) over a 15-month period, which precludes definitive statistical conclusions regarding the effectiveness of HEMS compared to traditional transport methods. As a pilot investigation, this study was not powered to detect differences in patient outcomes; its primary value lies in characterizing the feasibility and challenges of establishing such a service. The absence of a control group (e.g., patients transported by ferry) further limits the ability to quantify the incremental benefit of HEMS. Therefore, our results should be interpreted as hypothesis-generating. They underscore the critical need for future research, including larger, prospective, controlled studies or registry-based analyses with longer follow-up periods, to robustly evaluate the impact of maritime HEMS on morbidity and mortality. The operational insights gained from this initial experience, particularly the identification of the highly variable onset-to-dispatch interval as a key bottleneck, will be instrumental in designing these future studies and optimizing the system.

## Data Availability

The raw data supporting the conclusions of this article will be made available by the authors, without undue reservation.

## References

[1] Brown JB Smith KJ Gestring ML Rosengart MR Billiar TR Peitzman AB . Comparing the air medical prehospital triage score with current practice for triage of injured patients to helicopter emergency medical services: a cost-effectiveness analysis. JAMA Surg. (2017) 840–847. doi: 10.1001/jamasurg.2017.4485 PMC5885923 29094162

[2] Liu WW Chen J Yan S Niu Z Wang SF. Experience and exploration of medical rescue and treatment training of maritime aviation rescue detachment. Chin J Naut Med Hyperbar Med. (2023) 30:249–51.

[3] Deeb A-P Teng CY Peitzman AB Billiar TR Sperry JL Lu L . Direct trauma center access by helicopter emergency medical services is associated with improved survival after severe injury. Ann Surg. (2023) 278:e840–7. doi: 10.1097/SLA.0000000000005812 36735480 PMC10397363

[4] Professional Committee Of Emergency Resuscitation And Disaster Medicine Of Chinese Medical Doctor A. Rescue and protection branch of china medical rescue association expert consensus group on aviation rescue M. Zhonghua Wei Zhong Bing Ji Jiu Yi Xue. (2024) 36:225–30. doi: 10.3760/cma.j.cn121430-20240111-00034 38538348

[5] Liu T Jiang W Zhang M Xiong S Chen L Chen X . Intracerebral hemorrhage: advances, knowledge gaps, and future directions. MedComm. (2020) 6:e70436. doi: 10.1002/mco2.70436 41159140 PMC12554790

[6] Hua X Liu M Wu S. Definition, prediction, prevention and management of patients with severe ischemic stroke and large infarction. Chin Med J. (2023) 136:2912–22. doi: 10.1097/CM9.0000000000002885 38030579 PMC10752492

[7] Rose C Ter Avest E Lyon RM. Fatigue risk assessment of a helicopter emergency medical service crew working a 24/7 shift pattern: results of a prospective service evaluation. Scand J Trauma Resusc Emerg Med. (2023) 31:72. doi: 10.1186/s13049-023-01143-4 37924156 PMC10623805

[8] Ter Avest E Griggs J Prentice C Jeyanathan J. Lyon RM. Out-of-hospital cardiac arrest following trauma: What does a helicopter emergency medical service offer? Resuscitation. (2019) 135:73–9. doi: 10.1016/j.resuscitation.2018.12.019 30597132

[9] Saviluoto A Jäntti H Holm A Nurmi JO. Does experience in prehospital post-resuscitation critical care affect outcomes? A retrospective cohort study. Resuscitation. (2021) 163:155–61. doi: 10.1016/j.resuscitation.2021.03.023 33811958

[10] Petrowski K Malkewitz CP Schöniger C Frank M Theiler L. Stress of emergency physicians during helicopter operations: impact of patients' diagnoses, severity of diagnoses, and physicians' work experience. BMC Emerg Meed. (2023) 23:20. doi: 10.1186/s12873-023-00786-x 36803306 PMC9942287

