# Supplementary material for: A pilot study of helicopter emergency medical services in the Zhoushan Archipelago: clinical experience and operational insights from 18 cases
Source: Front Public Health. 2026 Aug 6;14:1834164. doi: 10.3389/fpubh.2026.1834164 (PMC13490067; doi:10.3389/fpubh.2026.1834164)
Supplement: Supplementary file 1 [file Table_1.docx]

Supplementary Table 1:Individual Patient Characteristics, Key In-flight Interventions, and Outcomes

| Patient | Primary Diagnosis / Injury | ISS | GCS | Key In-flight Interventions | Outcome |
| --- | --- | --- | --- | --- | --- |
| 1 | Severe Traumatic Brain Injury, Brain Herniation | 36 | 5 | Spinal Immobilization, Airway Management, Mannitol | Improved at Discharge* |
| 2 | Traumatic Renal Rupture, Hemorrhagic Shock | 24 | 15 | Volume Resuscitation, Analgesia, Splinting | Improved |
| 3 | Ventricular Fibrillation / Cardiac Arrest | N/A | 3 | Endotracheal Intubation, Intravenous Access | Recovered |
| 4 | Spontaneous Cerebral Hemorrhage | N/A | 8 | Airway Management, Intravenous Access | Improved |
| 5 | Acute Coronary Syndrome | N/A | 15 | Post-thrombolysis Transfer, Intravenous Access | Recovered |
| 6 | Spontaneous Cerebral Hemorrhage | N/A | 15 | Intravenous Access, Blood Pressure Control | Improved |
| 7 | Multiple Upper Limb Fractures | 5 | 15 | Intravenous Access, Hemostasis, Limb Fixation | Transferred (to another hospital) |
| 8 | Spontaneous Cerebral Hemorrhage | N/A | 15 | Intravenous Access | Improved |
| 9 | Spontaneous Cerebral Hemorrhage | N/A | 15 | Intravenous Access | Improved |
| 10 | Acute Coronary Syndrome | N/A | 15 | Medication (ACS bundle), Intravenous Access | Recovered |
| 11 | Lower Limb Injury | 1 | 15 | Intravenous Access, Wound Care | Recovered |
| 12 | Traumatic Brain Hemorrhage | 11 | 15 | Intravenous Access, Hemostasis of Head Wound | Recovered |
| 13 | Lower Limb Injury | 2 | 15 | Intravenous Access, Wound Care | Recovered |
| 14 | Multiple Trauma (Severe Upper Limb Injury) | 14 | 15 | Intravenous Access, Hemostasis, Limb Fixation | Improved |
| 15 | Acute Coronary Syndrome | N/A | 15 | Medication (ACS bundle), Intravenous Access | Recovered |
| 16 | Acute Coronary Syndrome | N/A | 15 | Post-thrombolysis Transfer, Intravenous Access | Recovered |
| 17 | Lumbar Vertebra Fracture | 3 | 15 | Intravenous Access, Pelvic Binding, Analgesia | Recovered |
| 18 | Lower Limb Fracture | 5 | 15 | Intravenous Access, Hemostasis, Limb Fixation | Recovered |
